# The spells of iatrogeny

**DOI:** 10.1007/s12471-023-01762-7

**Published:** 2023-02-02

**Authors:** A. F. Cardoso, G. Dias, B. Faria, F. Almeida, A. Lourenço

**Affiliations:** Department of Cardiology, Hospital Senhora da Oliveira—Guimarães, Guimarães, Portugal

## Answer

**Fig. 1:**
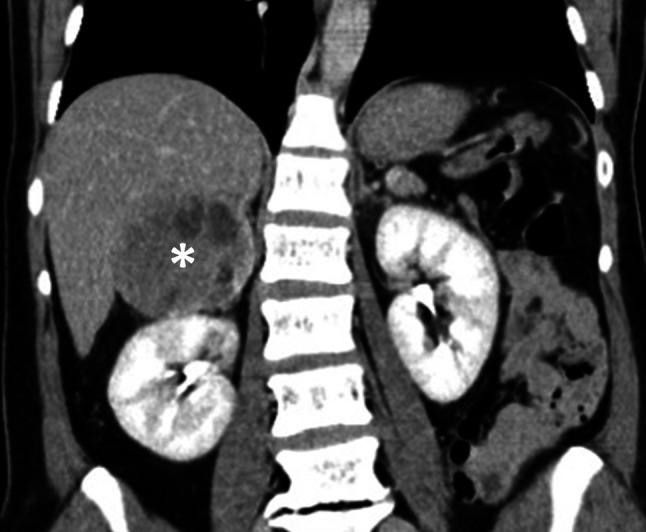
Computed tomography coronal view confirming presence of right adrenal mass (denoted with *asterisk*)

The paroxysms of tachycardia and arterial hypertension, clinical presentation and sudden worsening after beta-blocker and metoclopramide therapy in this patient raised the suspicion of a pheochromocytoma. Urgent thoracoabdominal computed tomography confirmed the presence of a right adrenal mass (size: 75 × 69 × 73 mm) (Fig. 1). Subsequently, beta-blocker therapy was withheld. Plasmatic and 24-hour urinary catecholamine and metanephrine levels were found to be markedly elevated.

Stepwise alpha-adrenergic blockade with phenoxybenzamine followed by beta-adrenergic blockade with propranolol were undertaken. Blood pressure and heart rate normalised, and her symptoms resolved. Left ventricular abnormalities recovered after 5 days, confirming Takotsubo-like cardiomyopathy. An uneventful right laparoscopic adrenalectomy was performed, and histological examination confirmed the diagnosis of pheochromocytoma. The patient remained asymptomatic and normotensive during follow-up.

Pheochromocytoma is a rare tumour with potentially life-threatening complications. High catecholamine levels can lead to direct cardiotoxicity, resulting in a Takotsubo-like cardiomyopathy presentation [1]. Pheochromocytoma paroxysms can be precipitated by certain conditions, namely exercise, trauma, surgery or specific drugs. The latter include beta-blockers, which promote significative unopposed alpha-mediated vasoconstriction, and metoclopramide, by potentiating additional catecholamine release [2]. In this case, these two drugs probably acted synergistically, leading to clinical worsening and uncovering the cardinal manifestations.

Pheochromocytoma can present with nonspecific symptoms, making this a challenging diagnosis that requires a high index of suspicion. Nevertheless, important red flags are paroxysmal hypertension, the triad of episodic headache, diaphoresis and palpitations, or clinical worsening under the previously mentioned conditions [3]. In patients with Takotsubo-like cardiomyopathy, active surveillance for these signs/symptoms is important as they can expose an unrecognized pheochromocytoma, allowing treatment and avoidance of potentially harmful medications.

## References

[1] Gupta S, Goyal P, Idrees S (2018). Association of endocrine conditions with Takotsubo cardiomyopathy: a comprehensive review. J Am Heart Assoc.

[2] Eisenhofer G, Rivers G, Rosas AL (2007). Adverse drug reactions in patients with phaeochromocytoma: incidence, prevention and management. Drug Saf.

[3] Neumann HPH, Young WF, Eng C (2019). Pheochromocytoma and Paraganglioma. N Engl J Med.

